# *Neisseria gonorrhoeae* Antimicrobial Resistance: The Future of Antibiotic Therapy

**DOI:** 10.3390/jcm12247767

**Published:** 2023-12-18

**Authors:** Angelo Roberto Raccagni, Martina Ranzenigo, Elena Bruzzesi, Chiara Maci, Antonella Castagna, Silvia Nozza

**Affiliations:** 1 Infectious Diseases Unit, Vita-Salute San Raffaele University, 20132 Milan, Italy; ranzenigo.martina@hsr.it (M.R.); bruzzesi.elena@hsr.it (E.B.); maci.chiara@hsr.it (C.M.); castagna.antonella1@hsr.it (A.C.); nozza.silvia@hsr.it (S.N.); 2 Infectious Diseases Unit, IRCCS San Raffaele Scientific Institute, 20132 Milan, Italy

**Keywords:** gonorrhoea, gonorrhea, drug resistance, antimicrobials, clinical trials, antibiotics, zoliflodacin, gepotidacin

## Abstract

The growing threat of antibiotic-resistant *Neisseria gonorrhoeae*, which causes gonorrhea, presents a current public health challenge. Over the years, the pathogen has developed resistance to different antibiotics, leaving few effective treatment options. High-level resistance to key drugs, including ceftriaxone, has become a concerning reality. This article primarily focuses on the treatment of gonorrhea and the current clinical trials aimed at providing new antibiotic treatment options. We explore ongoing efforts to assess new antibiotics, including zoliflodacin, and gepotidacin. These drugs offer new effective treatment options, but their rapid availability remains uncertain. We delve into two ongoing clinical trials: one evaluating the efficacy and safety of gepotidacin compared to the standard ceftriaxone–azithromycin combination and the other assessing the non-inferiority of zoliflodacin versus the combination therapy of ceftriaxone–azithromycin. These trials represent crucial steps in the search for alternative treatments for uncomplicated gonorrhea. Notably, gonorrhea has been included in the “WHO Priority Pathogens List for Research and Development of New Antibiotics”. In conclusion, the urgent need for innovative treatment strategies is underscored by the rising threat of antibiotic resistance in *N. gonorrhoeae*; collaboration among researchers, industries, and healthcare authorities is therefore essential.

## 1. Introduction

Sexually transmitted infections (STIs) are a global public health challenge, severely affecting the quality of life and giving rise to substantial morbidity and mortality. STIs have a negative influence on the reproductive and child health, leading to complications such as infertility and pregnancy-related issues [1]. Most STIs facilitate the transmission of HIV, amplifying their impact on both national economies and the individual well-being [1]. Prioritizing STIs prevention and control is a paramount public health concern [2]. Curable STIs alone result in the loss of over eleven million disability-adjusted life years annually, and it is estimated that more than a million STIs are contracted daily worldwide, with infections including chlamydia, gonorrhea, syphilis, trichomoniasis, herpes simplex virus (HSV), human papillomavirus (HPV), and HIV falling under the broad category of these infections [2,3,4,5]. More than 1 million STIs every day are acquired. As reported by the WHO, in 2020, 374 million new infections with one of four STIs occurred. Chlamydia cases totaled 129 million, gonorrhea totaled 82 million, syphilis totaled 7.1 million and trichomoniasis totaled 156 million. Over 490 million people were believed to have genital herpes in 2016, and 300 million women were believed to have HPV infection.

## 2. Gonorrhea

Gonorrhea is the second most common bacterial STI inflicting considerable morbidity and substantial economic costs on a global scale [6]. Gonococci success as human pathogens is the result of several combined factors. The plethora of bacterial virulence factors allow efficient colonization of both male and female mucosae. The chameleon-like ability determined by the high-frequency antigenic and phase variation of core surface structures causes clonal variability. The ability to subvert and hide from the immune system by directly suppressing and interacting with immunological effectors and regulators allows re-infections. *N. gonorrhoeae* infections encompass a multi-step strategic process: pathogen highly efficient transmission; mucosal localized adherence; local proliferation and invasion; local inflammatory response; external or systemic dissemination. Presently, there is no effective vaccine directly targeting *Neisseria gonorrhoeae* available in clinical practice, although data support the possible use of multicomponent meningococcal serogroup B (4CMenB) vaccine to provide some cross-protection and also prevent *Neisseria gonorrhoeae* cases [2,3,4,5,6,7]. With the emergence of multidrug-resistant (MDR) strains and the lack of new antibiotics, the World Health Organization (WHO) has recognized the health sector’s response to the epidemic of STIs as a critical component in achieving universal health coverage [8]. This objective is delineated in the 2030 Agenda for Sustainable Development, encapsulating the Sustainable Development Goals (SDGs) [9,10]. However, the sustainable control of infections may not be reached with the current interventions, necessitating also innovative solutions.

## 3. Epidemiology

In 2016, eighty-seven million new cases of *Neisseria gonorrhoeae* were estimated worldwide, with varying prevalence and incidence across the world and higher risk among younger individuals [11]. The highest rates were observed in low and middle-income countries, as transmission is often linked to the socio-economic status. Notably, gonorrhea prevalence among women was high in the WHO African region, the Americas, and the Western Pacific, while lower prevalence was observed in Europe. However, an increase in cases in Western countries is currently observed, which can be attributed to improved diagnostics and evolving sexual networks [11,12]. Moreover, in the African region, *Neisseria gonorrhoeae* during pregnancy poses a substantial burden, reaching a prevalence comparable to that of malaria [13]. Focusing on Europe, an increasing trend in cases is observed among all key populations with the most significant rise observed among men who have sex with men (MSM) [14,15,16,17]. Moreover, international travels play a key role in the spread of multi-resistant STIs, and therefore, improved surveillance in international travelers is needed. However, reported data are likely underestimated due to underreporting and the presence of asymptomatic cases. The bacterium’s emergence as an MDR pathogen has led to its inclusion in the “WHO Priority Pathogens List for Research and Development of New Antibiotics” [10]. Remarkably, *N. gonorrhoeae* is the only STI in the list; the second level of priority [10]. Indeed, the “World Health Organization Global Gonococcal Antimicrobial Surveillance Program,” known as the WHO GASP, is designed to monitor the emergence of MDR and extensively drug-resistant (XDR) strains [18]. The WHO recommends discontinuing first-line treatments when failures or not susceptible isolates are above 5%. Data from 2015 to 2016 show that 100% of countries reported ciprofloxacin resistance, 80% reported resistance to azithromycin, 45% reported resistance to cefixime and 24% reported resistance to ceftriaxone [18]. Ceftriaxone resistance is rare in the Euro-GASP countries when compared to other regions, whilst azithromycin resistance is highly prevalent also in the European region, although travel-related spread of MDR lineages is observed [18]. Moreover, the WHO established the Global Antimicrobial Resistance Surveillance System (GLASS) in 2019 [19].

## 4. Clinical Presentation

Gonorrhea infections involve the genital, rectal, and pharyngeal regions and may be either asymptomatic or symptomatic. Most acute lower tract infections in females, around 80–90% of cases, are asymptomatic, while lower tract infections in men are symptomatic in approximately 45% of cases. Extra-genital gonococcal infections are often asymptomatic among both men and women [3,11]. Complications can arise from both symptomatic and asymptomatic infections. The most common adverse outcomes are related to sexual and reproductive health and include upper genital tract dissemination, leading to pelvic inflammatory disease (PID), Fitz Hugh Curtis syndrome, tubal-ovarian abscess, epididymo-orchitis, bartholinitis, penile lymphangitis, and edema [11,20]. Genital tract scarring and adverse pregnancy outcomes also contribute to gonorrhea morbidity [11,20,21,22,23]. Rarely, gonorrhea may also present as disseminated gonococcal infection (DGI), which include gonococcemia, gonococcal arthritis, tenosynovitis, osteomyelitis, endocarditis, and meningitis [24,25]. Lastly, gonorrhea increases the risk of HIV acquisition, transmission, with often presence of co-infection with other STIs [26,27].

## 5. Diagnosis and Testing

Gonorrhea diagnosis should combine clear clinical signs followed by appropriate microbiological methods for confirmation of infection. However, especially among women, higher asymptomatic infection rates are observed. Asymptomatic individuals, especially young women, MSM, and people living with HIV (PLWH), should therefore undergo regular screenings along with comprehensive STI and HIV testing. Furthermore, all sexual partners of confirmed cases should be tested and treated to avoid risk of re-infection and given the high contagiousness of the bacterium [11,28]. Microscopy is useful for suspect gonorrhea in symptomatic men but less sensitive for cervical and extra-genital infections. However, microscopy is not gold standard and requires confirmation by means of culture or molecular testing [29]. Culture offers reliable sensitivity (between 85 and 95%) and assesses the antimicrobial susceptibility, making it valuable in cases of antibiotic resistance or treatment failure [11]. Nucleic Acid Amplification (NAAT) is the gold-standard exam, as it provides high sensitivity (over 90%) and specificity. NAAT can be performed on various samples, offering rapid results, but these do not include an evaluation of antimicrobial susceptibility [11,30].

## 6. Therapy

Over time, the recommended antibiotic therapy for *Neisseria gonorrhoeae* has evolved. In this section, we explore the latest guidelines, including those from the WHO and the Centers for Disease Control and Prevention (CDC). It is important to note that individuals with both gonorrhea and living with HIV should receive the same treatment of those living without HIV [11,31].

### 6.1. WHO Guidelines

The WHO issued guidelines in 2016 for managing uncomplicated gonorrhea infections [11]. They recommend dual therapy as the primary treatment regimen, although considering local resistance data is crucial. Dual therapy options include ceftriaxone in combination with azithromycin and cefixime in combination with azithromycin. For cases where an antibiogram or local resistance data are available, single therapy can be considered, involving ceftriaxone, cefixime and spectinomycin. However, for cases of gonococcal oropharyngeal infections, dual therapy is strongly recommended due to a higher incidence of treatment failures following single-drug treatment [11]. Notably, kanamycin and gentamicin were not yet recommended as the primary treatment due to limited available data. Indeed, gentamycin showed inferiority in clinical trials compared to first-line treatment options, and therefore, its use should be limited as a second-line option for treatment failures or the presence of allergy [32]. Although guidelines currently recommend cefixime as a possible treatment option, recently, a clinical trial investigated cefixime use for the treatment of gonorrhea. Among 161 enrolled volunteers receiving either cefixime and doxycycline or ceftriaxone and azithromycin, the combination of cefixime and doxycycline showed high efficacy for urogenital and rectal gonorrhea. However, it did not achieve non-inferiority for treatment of gonorrhea when including pharyngeal gonorrhea [33]. Although not currently recommend by international guidelines, gemifloxacin in combination with azithromycin might be another second-line treatment option for uncomplicated gonorrhea based on clinical trials results [34].

### 6.2. CDC Guidelines

The Centers for Disease Control and Prevention updated their 2015 STIs guidelines in 2020 in response to the rise of in MDR isolates and documented treatment failures and confirmed the statements in the 2021 guidelines, suggesting new first-line treatment regimens with ceftriaxone in combination with doxycycline [35]. Alternative regimens include gentamicin and cefixime. To address the concerning increase in high-level azithromycin resistance, the Centers for Disease Control and Prevention removed azithromycin from empirical therapy and doubled the ceftriaxone dosage from 250 to 500 mg. Indeed, there is high debate among physicians regarding whether gonorrhea treatment should, in the absence of chlamydia, consist only of single therapy with ceftriaxone. This would preserve azithromycin use, given that an increase in minimum inhibitory concentrations (MICs) is observed worldwide and its widespread use for respiratory tract infections. Given the common co-infection of *N. gonorrhoeae* and *C. trachomatis*, and the rise of treatment failures with azithromycin when treating chlamydia, this dual therapy is more effective against both bacteria [11,35]. These recommendations find support in pharmacokinetic and pharmacodynamics principles. Ceftriaxone is a bactericidal, time-dependent antibiotic, meaning its efficacy is closely tied to the duration during which its serum concentration remains above the MIC of the bacteria. An MIC exceeding 0.125 mcg/mL signals potential ceftriaxone-resistant strains. A 250 mg ceftriaxone dose fails to maintain a concentration above the MIC for an extended period, whereas 500 mg dosages ensure a more extended free-drug duration beyond 24 h. Treatment failures in gonorrhea are most observed in oropharyngeal infections, which can be explained by the wide variability in ceftriaxone concentrations in this anatomical site. Effective treatment in such cases requires longer exposure above the MIC, making higher antimicrobial dosages highly recommended. Moreover, it should be noted that ertapenem was also found in a recent clinical trial to be an effective alternative regimen for anogenital gonorrhea that is non-inferior to ceftriaxone [32]. This treatment option might play a role in the treatment of drug-resistant strains or in case of the presence or suspected of allergies to cephalosporins [32]. However, it should be noted that therapy with carbapenems cannot be applied to routine clinical practice or be regarded as a first-line treatment options given that these drugs should be preserved and spared for the treatment of other MDR infections and in order to avoid further emergence of resistant strains, especially among Gram-negative bacteria.

## 7. Antimicrobial Resistance

Ever since the introduction of antibiotic therapy for gonorrhea, the bacterium *N. gonorrhoeae* has been steadily developing high-level resistance to a wide range of drug classes [11,31]. The emergence of MDR *N. gonorrhoeae* strains is caused by a variety of factors. Notably, the over-prescription of antimicrobials has played a significant role in driving this trend [11]. Furthermore, the bacterium’s natural competence for transformation is an additional complicating factor [36]. Gonococci exhibit a remarkable ability to acquire exogenous DNA from other *Neisseriae*, facilitating the incorporation of foreign genetic material into their own genome [36]. This concerning trend is reflected in the estimated resistance of over half of clinical specimens to at least one antibiotic class. The CDC warns that 550,000 cases of drug-resistant gonorrhea occur annually out of the 1.14 million total reported cases [12]. Even first-line antibiotics like ceftriaxone are not immune to the problem of decreasing susceptibility and emerging resistance (DS/R) [11]. Notably, instances of high-level resistance to ceftriaxone and azithromycin were first documented in England and Australia in 2018 [37,38]. Furthermore, between 2015 and 2019, documented treatment failures with the current recommended dual therapy regimen were observed for pharyngeal infections [11,31,37,38]. Cases of extensive drug-resistant XDR specimens have been reported in various parts of the world, including France, Spain, and Japan [11,31]. Specific core groups that are particularly prone to repeated *N. gonorrhoeae* infections experience higher rates of resistance [39]. This phenomenon is not only attributed to high-risk sexual behaviors but also to the elevated prevalence of resistant isolates in MSM compared to men who have sex with women, as in these distinct sexual networks, gonococcal strains with antibiotic resistance tend to circulate more rapidly [3,37,39,40]. Factors which might limit the increase in antimicrobial resistance include infection prevention, also by means of vaccines and pharmacological approaches, extensive screening and rapid treatment, and the development of novel antibiotics. Given the recent evidence on the possibility to use doxycycline post-exposure prophylaxis (doxyPEP) against bacterial STIs (especially syphilis and chlamydia) following sexual exposure, it must be taken into account that tetracycline resistance is very common among gonococci strains, therefore rendering this prophylaxis scarcely effective against gonorrhea [41].

## 8. Novel Antibiotics Showing Inferiority to First-Line Treatment Options

The demand for effective new treatment options for gonorrhea has been a persistent challenge marked by several attempts to address the evolving landscape of antimicrobial resistance. Despite efforts of researchers and pharmaceutical companies, some promising clinical trials failed to demonstrate the non-inferiority of investigational products when compared to the existing first-line treatment options for gonorrhea.

### 8.1. Solithromycin

Solithromycin is a broad-spectrum fluoro-ketolide which was evaluated in clinical trials for the treatment of gonorrhea. It exhibited high activity against most gonococcal strains, including drug-resistant ones. However, its progress for gonococcal treatment was interrupted due to its failure to demonstrate non-inferiority compared to the ceftriaxone [42,43,44]. Overall, 261 volunteers were treated with either solithromycin or ceftriaxone and azithromycin. Of these, 80% in the solithromycin group and 84% in the combination group achieved gonorrhoeae eradication. The difference was not significant enough to conclude that solithromycin was non-inferior to standard of care. The solithromycin group also experienced more frequently adverse events, as diarrhea and nausea. Therefore, solithromycin as a single 1000 mg dose is not recommended as a first-line treatment for gonorrhea [44].

### 8.2. Delafloxacin

Delafloxacin is a novel broad-spectrum fluoroquinolone with increased potency and target affinity compared to older fluoroquinolones. Studies showed activity against MDR *N. gonorrhoeae*. However, delafloxacin was found in a clinical trial to not be a reliable treatment for urogenital gonorrhea given the high rate of observed treatment failures. Overall, 460 participants were randomly assigned to receive either 900 mg of oral delafloxacin or ceftriaxone. Delafloxacin had a urogenital cure rate of 85% compared to 91% of ceftriaxone, not meeting the non-inferiority margin [45].

## 9. New Antibiotics Candidates

Promising antimicrobial agents in advanced clinical development for gonorrhea treatment include zoliflodacin and gepotidacin. Following discussion on the rationale for use of these novel treatments, we here present insight on the ongoing clinical trials and their design. In order to investigate this aspect, we conducted systematic research on Clinical Trials.org regarding ongoing interventional clinical trials using key terms “Gonorrhea; Gonorrhoea; Gonococcal; *Neisseria gonorrhoeae*; Gonococcal infection; *Neisseria gonorrheae* infection; Gonococcal infections”. Completed studies with results available were discussed above; clinical trials actively investigating new drug candidates without available results are hereby presented.

### 9.1. Zoliflodacin

Zoliflodacin, a novel topoisomerase inhibitor, effectively inhibits bacterial DNA biosynthesis, making it useful for treating uncomplicated gonorrhea. Its distinct mode of action suggests potential efficacy also against fluoroquinolone-resistant strains [43,46,47,48,49,50]. Phase II trials demonstrated its safety and tolerability among subjects with gonorrhea.

Participants were randomly assigned to receive a single oral dose of either 2 g or 3 g of zoliflodacin or ceftriaxone. The primary outcome measure was the proportion of urogenital microbiologic cure. Microbiologic cure at urogenital sites was documented in 96% of participants who received 2 g of zoliflodacin, 96% who received 3 g, and 100% who received ceftriaxone. Reported adverse events were mostly gastrointestinal. Zoliflodacin showed promise in treating urogenital and rectal gonococcal infections but was less effective for pharyngeal infections. This limitation is consistent with previous recommendations for other drugs, such as spectinomycin and other fluoroquinolones [46].

### 9.2. Gepotidacin

Gepotidacin, a triazaacenaphthylene antibiotic, shows bactericidal effects by inhibiting the DNA topoisomerase II activity. It demonstrated low MIC values against various gonococcal strains, including ciprofloxacin-resistant ones. Combination studies with other antibiotics showed also a synergistic effect. A phase II trial indicated a 95% success rate in treating uncomplicated genitourinary gonorrhea [42,51,52,53]. Overall, 69 participants received either a 1500 mg or 3000 mg single oral dose of gepotidacin. Microbiological eradication of the infection was achieved by 97% and 95% of participants in the 1500 mg and 3000 mg groups, respectively, without treatment-limiting adverse events. A single, oral dose of gepotidacin was indeed highly effective for the treatment of uncomplicated urogenital infections [53].

## 10. Ongoing Phase III Interventional Clinical Trials

### 10.1. A Study Evaluating Efficacy and Safety of Gepotidacin Compared with Ceftriaxone plus Azithromycin in the Treatment of Uncomplicated Urogenital Gonorrhea

This clinical trial represents a phase III study, conducted at multiple research centers, and follows an open-label, randomized design. Its primary objective is to assess the efficacy and safety of oral gepotidacin in comparison to a treatment regimen involving intramuscular (IM) ceftriaxone combined with oral azithromycin. The trial focuses on the treatment of uncomplicated urogenital infections caused by *N. gonorrhoeae* and includes both adolescent and adult participants. Participants will be randomly assigned to one of two groups, with one group receiving oral gepotidacin and the other group receiving the combination of IM ceftriaxone and oral azithromycin. The primary outcome measure is the number of participants achieving culture-confirmed bacterial eradication of *N. gonorrhoeae* from the urogenital site at the test of cure (TOC). This entails obtaining a pre-treatment urogenital swab specimen for bacteriological culture for *N. gonorrhoeae*, which is followed by assessing culture-confirmed bacterial eradication within 4 to 8 days post-treatment. Participants will receive gepotidacin orally at the study site during the baseline (Day 1) visit. Following this, they will self-administer a second oral dose as outpatients, typically 10 to 12 h after the first dose. Gepotidacin will be administered in the form of a 3000 mg oral dose (four 750 mg tablets) at the study site with each dose taken after food consumption and accompanied by water. Participants in the comparator group will receive a single intramuscular dose of 500 mg of ceftriaxone as well as a single oral dose of 1000 mg of azithromycin at the study site during the baseline (Day 1) visit [54].

### 10.2. A Multi-Center, Randomized, Open-Label, Non-Inferiority Trial to Evaluate the Efficacy and Safety of a Single, Oral Dose of Zoliflodacin Compared to a Combination of a Single Intramuscular Dose of Ceftriaxone and a Single Oral Dose of Azithromycin in the Treatment of Patients with Uncomplicated Gonorrhea

This clinical trial represents a phase III, non-inferiority study that is multi-centered and openly conducted. It aims to evaluate the safety and effectiveness of a 3 g oral dose of zoliflodacin in comparison to a combination therapy involving a single 500 mg intramuscular dose of ceftriaxone and a single 1 g oral dose of azithromycin for the treatment of uncomplicated cases of gonorrhea. Participants in the experimental group will receive a single 3 g oral dose of zoliflodacin, and the dose will be administered orally. Participants in the comparator group will receive a combination therapy consisting of a single 500 mg intramuscular dose of ceftriaxone and a single 1 g oral dose of azithromycin. The primary outcome measure of this trial is to assess the efficacy of a single dose of zoliflodacin compared to the combination therapy of a single dose of ceftriaxone and azithromycin. This assessment will involve the microbiological testing of cultures from urethral or cervical sites during the cure test visit, which is scheduled for day six post-treatment [55]. Moreover, in order to grant further insight on gepotidacin pharmacokinetics and potential use for prostatic infections and pharyngeal gonorrhea treatment, another study is investigating the drug concentrations in plasma, prostate and tonsillar tissue of patients undergoing prostatectomy or tonsillectomy [56].

## 11. Discussion

The emergence and rapid spread of antimicrobial resistance in *N. gonorrhoeae* presents a significant and growing public health challenge, which occupies the second level of priority in the WHO list of drug-resistant pathogens. Over the years, *N. gonorrhoeae* has developed resistance to multiple classes of antibiotics, leaving few effective treatment options for treatment of drug-resistant strains. This resistance trend is alarming, with over half of clinical specimens displaying resistance to at least one antibiotic class, and high-level resistance to key drugs such as ceftriaxone becoming a concerning reality worldwide. The mechanisms underlying this resistance are complex and involve alterations in antibiotic targets, efflux pumps, and the expression of resistance-conferring enzymes. Several factors, including the over-prescription of antibiotics and the bacterium’s ability to acquire exogenous genetic material, have contributed to the development of multidrug-resistant strains. One of the challenges appears to be developing a novel antimicrobial which could be used to tackle emergence to drug resistance, with high effectiveness against pharyngeal gonorrhea, which represents the anatomical site with the most frequent treatment failures due to pharmacokinetics issues. To date, trials have already established that we have antibiotics which might be effective in case of ceftriaxone resistance, for instance carbapenems. However, to avoid the over-prescription of these drugs and avoid the further emergence of antimicrobial resistance especially among Gram-negative bacteria, their use is likely not applicable to routine clinical care and as first-line regimens. Their use should be limited to cases of documented treatment failure without alternative treatment options or in case of documented allergy to other treatment options. Therefore, novel drugs are urgently needed, possibly with a convenient oral route administration. Although in the past, several drug candidates have failed to demonstrate non-inferiority to current first-line regimens, to combat this challenge, promising new antibiotic candidates are in various stages of clinical development. Most importantly zoliflodacin and gepotidacin appear to be in late stages of clinical research. These drugs offer hope for effective treatment, but their availability remains uncertain in a very short-term scenario. Ongoing clinical trials are evaluating the efficacy and safety of these new antibiotics, which given that both imply an oral administration would be far more convenient than current intramuscular ceftriaxone, for instance when referring to low-income countries or in case of limited access to healthcare facilities. In conclusion, the escalating threat of antibiotic resistance in *N. gonorrhoeae* underscores the urgency of action. The need for innovative treatment strategies, improved surveillance, and the development of new tools, also including vaccines, is paramount to address this healthcare challenge. The prevention of new infections, by means of pharmacological and non-pharmacological tools, along with effective testing and treatment strategies, also by means of novel antibiotics, might ultimately allow the containment of new infections and the resulting morbidity. Collaboration among researchers, industries, and healthcare authorities is therefore essential in order to develop new antibiotics, which should be linked to other key issues in STIs such as the development of global targets, prevention, testing and treatment policies, monitoring drug resistance and setting the global research agenda on STIs.

## Data Availability

Not applicable.
